# Promising Druggable Target in Head and Neck Squamous Cell Carcinoma: Wnt Signaling

**DOI:** 10.3389/fphar.2016.00244

**Published:** 2016-08-12

**Authors:** Amnani Aminuddin, Pei Yuen Ng

**Affiliations:** Drug Discovery and Development Research Group, Faculty of Pharmacy, Universiti Kebangsaan MalaysiaKuala Lumpur, Malaysia

**Keywords:** canonical Wnt signaling, β-catenin, cancer stem cells, head and neck squamous cell carcinoma, druggable target

## Abstract

Canonical Wnt signaling pathway, also known as Wnt/β-catenin signaling pathway, is a crucial mechanism for cellular maintenance and development. It regulates cell cycle progression, apoptosis, proliferation, migration, and differentiation. Dysregulation of this pathway correlates with oncogenesis in various tissues including breast, colon, pancreatic as well as head and neck cancers. Furthermore, the canonical Wnt signaling pathway has also been described as one of the critical signaling pathways for regulation of normal stem cells as well as cancer cells with stem cell-like features, termed cancer stem cells (CSC). In this review, we will briefly describe the basic mechanisms of Wnt signaling pathway and its crucial roles in the normal regulation of cellular processes as well as in the development of cancer. Next, we will highlight the roles of canonical Wnt signaling pathway in the regulation of CSC properties namely self-renewal, differentiation, metastasis, and drug resistance abilities, particularly in head and neck squamous cell carcinoma. Finally, we will examine the findings of several recent studies which explore druggable targets in the canonical Wnt signaling pathway which could be valuable to improve the treatment outcome for head and neck cancer.

## Introduction

Head and neck squamous cell carcinoma (HNSCC) is the most frequent type of head and neck cancer that usually arises from the mucosal surfaces of several organs including nasal cavity, paranasal sinuses, oral cavity, tongue, pharynx, and larynx (Sengupta, 2012). Globally, it is the sixth most common type of cancer (Jemal et al., 2010; Szafarowski and Szczepanski, 2013) with low survival rates due to late stage diagnosis and high rates of recurrence (Patel et al., 2014). Current therapeutic approaches in treating HNSCC patients are limited in choice and efficacy, hampered by physiological side effects, as well as risks of recurrence (Progress et al., 2008; Chen et al., 2011). The current understanding of recurrence is due to the existence of a subpopulation of cancer cells which are resistant to the mainstream therapy that focuses on eliminating the bulk tumor. This critical subpopulation of cells, also known as cancer stem cells (CSC), exhibits stem cell-like properties including self-renewal and drug- and radio- resistance capacities (Mannelli and Gallo, 2012; Routray and Mohanty, 2014). This CSC theory creates insight into the possibility of new therapeutic approaches for HNSCC. Exploring and understanding the molecular mechanisms underlying the therapeutic resistance, self-renewal, tumorigenicity and metastasis abilities of these CSC is important for the discovery of promising druggable targets in HNSCC.

Recently, it has been proposed that the aberrant activation of canonical Wnt signaling pathway in CSC is one of the underlying mechanisms of the progression of cancers as demonstrated in several cancer studies such as colon (Vermeulen et al., 2010), lung (Zhang et al., 2015), breast (Lamb et al., 2013) as well as head and neck cancer. Therefore, this review will highlight the role of canonical Wnt signaling pathway in the regulation of the properties of CSC, particularly in HNSCC. Thorough understanding of this specific Wnt signaling pathway may provide future insight for targeting CSC through the pathway for new therapeutic interventions intended to reduce the risk of tumor recurrence in HNSCC.

## Stem Cells, Cancer Stem Cells and Their Properties

Stem cells can be classified as a group of cells which are able to self-renew through indefinite cell division over a long period of time and have the potential to differentiate into several specific cell types. They do not perform any specialized function in the body as they do not have any tissue-specific structures. When triggered by either intrinsic or extrinsic signals, they undergo cell division to produce daughter cells where each daughter cell could remain as a stem cell or become a progenitor cell that further differentiates into a mature cell with more specialized functions (Fuchs and Segre, 2000; Reya et al., 2001; Mannelli and Gallo, 2012). In general, there are two types of human stem cells, namely the embryonic and adult stem cells. Embryonic stem cells are derived from the inner cell mass of blastocyst at the very early stage of embryo. They are pluripotent cells which can differentiate into any different types of cells in the human body excluding extra embryonic structures such as placenta and yolk sac (Hall, 1989). Meanwhile, adult stem cells, also known as tissue-specific stem cells, can be found in postnatal tissues and give rise into one or many but not all cell types of organs or tissues in which they reside (Hall, 1989; Schepers, 2003; Shigeo, 2005). Thus, adult stem cells are classified as unipotent or multipotent stem cells.

Recently, the theory of CSC has rapidly evolved owing to the first discovery of similarities in the stemness properties between normal hematopoietic stem cells (HSC) and a rare population of cells isolated from acute myeloid leukemic (AML) cells. In xenotransplantation of immunodeficient mice, these stem-like cells, termed severe combined immunodeficiency disease leukemia-initiating cells (SL-IC), proliferate, differentiate, and eventually initiate AML, a disease that was identical to the original human donor. These SL-IC also exhibit a remarkable self-renewing capacity whereby they promote reestablishment of AML in a secondary recipient mouse injected with SL-IC derived from the primary immunodeficient mice (Bonnet and Dick, 1997). Following the discovery, subsequent studies have uncovered the existence of stem-like cells in many other cancers including solid tumors such as breast (Al-Hajj et al., 2003), brain (Singh et al., 2003), prostate (Collins et al., 2005), pancreatic (Li et al., 2007), lung (Eramo et al., 2008), and head and neck tumors (Prince et al., 2007).

Generally, the regulatory mechanisms of stem cell features for both normal and CSCs are mainly associated with the homeostatic regulation by neighboring cells named niche, which provides a specialized microenvironment by generating extrinsic signals to regulate stem cell proliferation and cell fate determination (Dzierzak and Enver, 2008). Several regulatory signaling molecules that have been extensively studied include Hedgehog, Wnt, Notch and bone morphogenetic proteins (BMP; Li, 2006). The balance between proliferative and anti-proliferative signals governs self-renewal of stem cells. It is proposed that uncontrolled proliferation in cancer could be due to intrinsic mutation where stem cells become self-sufficient for growth or alterations in the niche which disrupt the balance of proliferative signals (Pardal et al., 2003; Li, 2006; Peerani et al., 2007). Meanwhile, the niche also provides balance between differentiation-inducing or -inhibiting signals for controlling cell fate (Peerani et al., 2007). Other than the basic features of self-renewal and differentiation, the niche may also regulate the properties of homing and mobilization of stem cells. Intriguingly, CSC exploit the same machinery that regulates normal stem cell homing and mobilization features for cancer invasion and metastasis (Li, 2006). Furthermore, normal stem cells such as HSC express drug-resistance proteins such as ATP-binding cassette (ABC) family transporter proteins to evade apoptosis induction for extended lifespan of the cells (Chaudhary, 1991). Remarkably, many recent studies demonstrated that CSC also express these drug-resistance proteins and hence explained the failure of current chemotherapy as well as the occurrence of cancer relapse (Cojoc et al., 2014).

## Wnt Signaling Pathway

Wnt signaling pathway is an important mechanism for embryonic development and homeostasis of mature tissues (MacDonald et al., 2009; Takahashi-Yanaga and Kahn, 2010; Nalbantoglu et al., 2011; Noguti et al., 2012; Novellasdemunt et al., 2015). It plays a vital role in controlling cell proliferation, differentiation, apoptosis, polarity, and migration (Kléber and Sommer, 2004; Katoh and Katoh, 2007; Vlad et al., 2008; MacDonald et al., 2009; Takahashi-Yanaga and Kahn, 2010; Nalbantoglu et al., 2011; Noguti et al., 2012). Wnt signaling pathway can be divided into two, namely canonical and non-canonical pathways. Both pathways are activated by the binding of Wnt protein to Frizzled (Fzd) seven transmembrane receptor. The fundamental difference between these two pathways is the involvement of β-catenin (Barker and Clevers, 2007; Katoh and Katoh, 2007; Curtin and Lorenzi, 2010; Takahashi-Yanaga and Kahn, 2010; Takebe et al., 2010; Le et al., 2015; Madan et al., 2015).

Non-canonical Wnt signaling pathways, which are independent of β-catenin, rely on the signal transduction of Wnt through Fzd as well as its coreceptors such as receptor tyrosine kinase-like orphan receptor 2 (ROR2) or receptor-like tyrosine kinase (RYK) (Rao and Kühl, 2010). The signal transduction will further activate intracellular scaffolding protein Dishevelled (Dvl)-dependent and/or calcium-dependent signaling cascades which are important for regulation of cell polarity, cytoskeletal rearrangements and cell movement (Giles et al., 2003; Katoh and Katoh, 2007). The Dvl-dependent signaling cascades include Rho family GTPases (such as RhoA and Rac) and c-jun N-terminal kinase (JNK) signaling cascades. RhoA activation is important for regulation of cytoskeletal rearrangements while activation of c-jun N-terminal kinase (JNK) signaling cascades through activated Rac is implicated in cell polarity control (Katoh and Katoh, 2007; Nalbantoglu et al., 2011). On the other hand, the calcium-mediated Wnt pathway involves the activation of heterotrimeric G-proteins upon Wnt binding to Fzd (Rao and Kühl, 2010). This will in turn activate phospholipase C (PLC), an important enzyme which promotes intracellular calcium release (Nalbantoglu et al., 2011). The increase in calcium level results in the activation of several signaling cascades such as JNK, nemo-like kinase (NLK) and nuclear factor of activated T (NFAT) cascades through calcium-responsive proteins including protein kinase C, calcium/calmodulin-dependent kinase II (CaMKII) and/or calcineurin (Rao and Kühl, 2010; Nalbantoglu et al., 2011; Anastas and Moon, 2013). Nuclear translocation of NFAT upon activation by calcineurin promotes expression of genes which are important for convergent extension during early embryogenesis (Li, 2006; Peerani et al., 2007). Besides modulating non-canonical Wnt signaling pathway, activated NLK also phosphorylates T-cell factor (TCF), an important transcription factor involved in the canonical Wnt signaling pathway. Phosphorylation of TCF inhibits the binding of β-catenin, a major mediator of canonical Wnt signaling pathway, to TCF and eventually leads to suppression of canonical Wnt signaling pathway (Katoh and Katoh, 2007; Rao and Kühl, 2010; Nalbantoglu et al., 2011). The crosstalk between these two signaling pathways clearly demonstrates the antagonistic mechanism between canonical and calcium-mediated non-canonical Wnt signaling pathways (Nalbantoglu et al., 2011).

**FIGURE 1:**
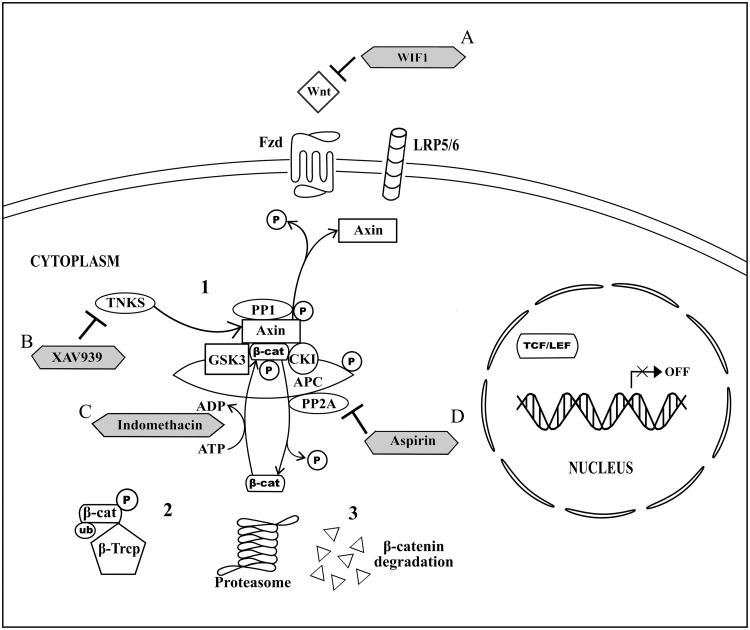
**Non-activated canonical Wnt signaling pathway and the drug targeted components.** (1) In the absence of Wnt signaling transduction, Axin, GSK3, CK1, and APC form a multiprotein destructive complex to promote β-catenin phosphorylation. In the negative feedback regulation, PP1 promotes dephosphorylation of Axin and subsequent disassembly of multiprotein destructive complex, whereas PP2A binds to Axin and/or APC and directs β-catenin dephosphorylation. (2) β-Trcp binds to the phosphorylated β-catenin for β-catenin ubiquitination. (3) Proteasomal degradation of the ubiquinated and phosphorylated β-catenin results in cytoplasmic β-catenin destruction. (A) WIF1 directly binds to Wnt for inhibition of Wnt binding to Fzd. (B) XAV939 inhibits activity of TNKS and thus promotes Axin upregulation. (C) Indomethacin promotes β-catenin degradation by phosphorylating β-catenin. (D) Aspirin acts as an inhibitor for negative feedback regulation modulated by PP2A and eventually directs β-catenin phosphorylation.

On the other hand, the canonical Wnt signaling pathway, also known as Wnt/β-catenin signaling pathway, involves the activation of cytoplasmic β-catenin signaling cascades upon Wnt signal transduction at the cell membrane (Logan and Nusse, 2004). Briefly, in the absence of Wnt (**Figure 1**), Axin-GSK3-CK1 complex is formed when GSK3 and CK1 bind to the separate domains of the Axin. Meanwhile, cytoplasmic β-catenin directly binds to adenomatous polyposis coli (APC; MacDonald et al., 2009). Both Axin-GSK3-CK1 complex and β-catenin-bounded APC form a multiprotein destructive complex to facilitate β-catenin phosphorylation (MacDonald et al., 2009). GSK3 and CK1 in the complex phosphorylate Axin and APC to increase the association of Axin and APC with β-catenin and hence stabilize the complex for further β-catenin phosphorylation (Ilyas, 2005; MacDonald et al., 2009). The phosphorylation of β-catenin at four specific serine and threonine residues by the complex is modulated by GSK3 and CK1. In brief, serine-45 of β-catenin in the complex will be first phosphorylated by CK1 and this event will be followed by GSK-mediated phosphorylation of β-catenin at threonine-41, serine-37, and serine-33 residues (Ilyas, 2005; Katoh and Katoh, 2007; Wu and Pan, 2010). The phosphorylated β-catenin will then be recognized by the E3 ubiquitin ligase β-transducin repeat containing protein (β-Trcp), for β-catenin ubiquitination and subsequently degradation by proteasome (Giles et al., 2003; Nalbantoglu et al., 2011; Yu and Virshup, 2014). In its negative feedback regulation, two serine/threonine phosphatase, protein phosphatase 1 (PP1) and protein phosphatase 2A (PP2A), which bind to Axin and/or APC, act via opposing the role of GSK3 and CK1. PP2A directly regulates β-catenin dephosphorylation (Giles et al., 2003; Logan and Nusse, 2004; MacDonald et al., 2009) whereas PP1 promotes dephosphorylation of Axin and results in disruption of GSK3-Axin binding, and eventually the disassembly of the multiprotein destructive complex (MacDonald et al., 2009).

**FIGURE 2:**
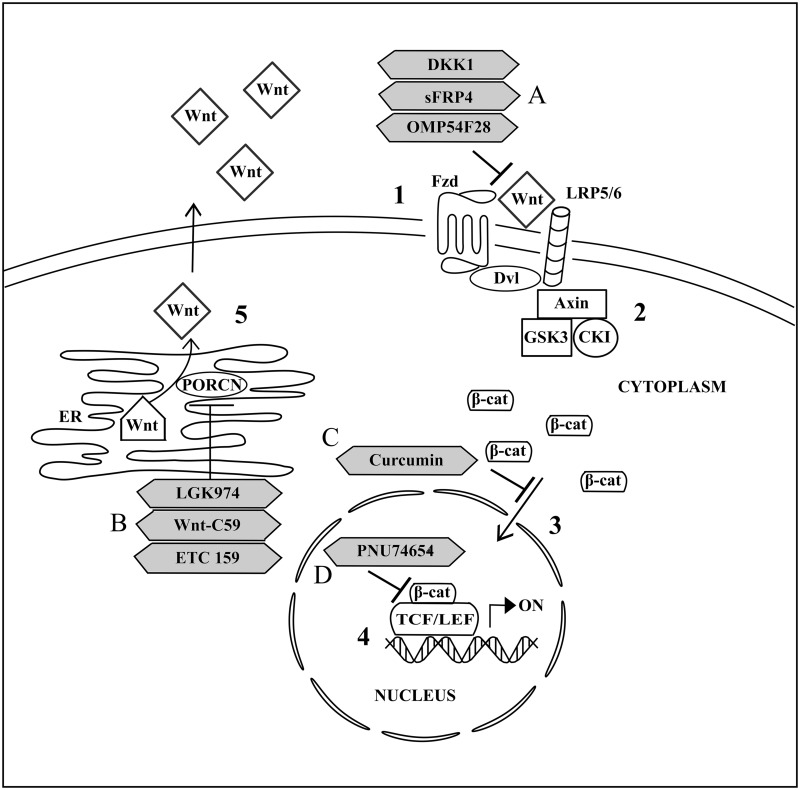
**Activated canonical Wnt signaling pathway and the drug targeted components.** (1) Signal transduction is activated upon binding of Wnt from outside the cell to Fzd and LRP 5/6 at the cell membrane. (2) Dvl and subsequently Axin, GSK3, CK1 are recruited to the cell membrane and thus inhibiting the formation of multiprotein destructive complex. (3) Accumulated cytoplasmic β-catenin is further translocated into nucleus. (4) Transcription of Wnt targeted genes is activated upon binding of nuclear β-catenin to TCF/LEF. (5) Wnt palmitoylation and secretion to outside of the cell is facilitated by PORCN in the endoplasmic reticulum (ER). (A) OMP54F28 and sFRP4 are antagonists for Wnt and Fzd interaction while DKK1 antagonizes Wnt/β-catenin signaling through binding to LRP 5/6. (B) LGK974, Wnt-C59, and ETC 159 are inhibitors for PORCN activities. (C) Curcumin acts as an inhibitor for nuclear translocation of cytoplasmic β-catenin. (D) PNU74654 functions as an inhibitor for interaction nuclear β-catenin with TCF/LEF.

In the presence of Wnt (**Figure 2**), the binding of Wnt to Fzd and its co-receptor, lipoprotein receptor-related protein 5 or 6 (LRP 5/6), leads to the formation of Wnt-Fzd-LRP 5/6 complex. This is followed by the recruitment of Dvl to Fzd (Takahashi-Yanaga and Kahn, 2010). This recruitment promotes LRP 5/6 phosphorylation and subsequently stimulates Axin-GSK3-CK1 complex recruitment to the Wnt-Fzd-LRP 5/6-Dvl complex at the cell membrane (Curtin and Lorenzi, 2010). The recruitment of Axin-GSK3-CK1 complex to the cell membrane prevents the binding of the complex to APC and β-catenin to form the functional multiprotein destructive complex for intrinsic kinase activity on β-catenin. Consequently, β-catenin will be accumulated in the cytoplasm and the increase in cytoplasmic β-catenin concentration will promote their translocation into the cell nucleus (Nalbantoglu et al., 2011). Nuclear β-catenin then binds to N-terminal of T-cell factor/lymphoid enhancing factor (TCF/LEF) transcription factors, leading to the transcription of Wnt targeted genes which are important for cellular development and maintenance (Kléber and Sommer, 2004; Chikazawa et al., 2010; Grumolato et al., 2010; Ota et al., 2012).

## Canonical Wnt Signaling Pathway in Cancer

Canonical Wnt signaling pathway acts as a master regulator for a wide range of biological effects through up- or down-regulation of genes that act as direct effectors, transcription regulators or other signaling pathway regulators. Thus, Wnt target genes expression can either directly or indirectly activate cell cycle progression, cell proliferation, cell differentiation, cell migration, inhibit apoptosis, and regulate embryonic development. Several examples of Wnt target genes are *CYCLIN D1*, *C-MYC*, cyclooxygenase 2 (*COX2*), bone morphogenetic protein 4 (*BMP4*), matrix metalloproteinases 7 (*MMP7*), *AXIN2*, multi-drug resistance 1 (*MDR1*), *β-TRCP*, and endothelial growth factor (*EGF*; Vlad et al., 2008).

Several studies have demonstrated that aberrant activation of canonical Wnt signaling pathway in several cancers can result from the upstream mutations of components of the pathway (**Table 1**). For instance, alteration in *CTNNB1*, a gene encoding β-catenin, causes prolonged half-life of β-catenin due to changes either at the APC recognition site or GSK3 phosphorylation site of β-catenin, by which both sites are important for degradation of β-catenin (Polakis, 2000; Giles et al., 2003). In hepatocellular carcinoma, mutation of *AXIN1*, which encodes for Axin, has been reported and this mutation causes an elevated level of β-catenin in the cytoplasm and nucleus of the cancer cells which eventually induces activation of β-catenin/TCF transcription (Satoh et al., 2000). Furthermore, mutation in genes encoding for several other components of the pathway including Wnt, Fzd, LRP 5/6, and APC, either causing loss or gain of protein function, have been reported in many cancers (MacDonald et al., 2009). For instance, sporadic colorectal cancer has been attributed to the alteration in gene encoding for APC (Nishisho et al., 1991). Other than that, mutation in gene encoding for Porcupine (PORCN), which is an endoplasmic reticulum resident enzyme involved in the process of Wnt secretion, has also been suggested to be one of the causes of focal dermal hypoplasia (Liu et al., 2013).

**Table 1:** Mutation in genes encoding for components of Wnt signaling pathway.

Gene	Encoded protein	Direct effect	Diseases	Reference
*CTNNB1*	Truncated β-catenin at APC and GSK3 recognition sites	Increased half-life of β-catenin	Colorectal cancer	Polakis, 2000; Giles et al., 2003
*AXIN1*	Truncated Axin with lack of β-catenin binding site	Accumulated β-catenin	Hepatocellular carcinoma	Satoh et al., 2000
*APC*	Loss-of-function APC	Accumulated β-catenin	Sporadic colorectal cancer	Nishisho et al., 1991
*PORCN*	Loss-of-function PORCN	Increased Wnt secretion	Focal dermal hypoplasia	Liu et al., 2013


The involvement of canonical Wnt signaling pathway in the formation of HNSCC has also been examined in several experimental studies. Evidence of the association between the pathway and HNSCC has been first discovered through cDNA arrays on patient samples. This study found that Fzd (GenBank Accession No. L37882), Fzd homolog 3 (FZD3; GenBank Accession No. U82169), and Dvl homolog (GenBank Accession No. U46461) genes, which are functionally important in the canonical Wnt signaling pathway, were highly expressed in HNSCC by two- to fivefold compared to normal tissues (Leethanakul et al., 2000). Furthermore, another study has also demonstrated that the gene expression levels of Wnts, particularly Wnt1 and Wnt10b, were markedly higher by 17- and 3-fold, respectively, in HNSCC cells compared to normal oral squamous epithelial cells (Rhee et al., 2002). Overexpression of these major components in HNSCC cells compared to normal cells clearly signifies the involvement of abnormal activation of Wnt signaling cascades in HNSCC.

Recently, canonical Wnt signaling pathway has also been described in the concept of stem cells. The fundamental role of the pathway in controlling stem cell expansion and differentiation has suggested that dysregulation of this stem cell regulatory pathway may result in cancer development. Several *in vitro* studies of breast, colon and head and neck cancers demonstrated that the pathway is highly activated in CSC compared to normal stem cells. For instance, overactivation of this pathway was demonstrated in breast tumor sphere growth, a method that enriches CSCs, compared to enriched normal breast stem cells (Lamb et al., 2013). The latter study showed an increase in expression of β-catenin and downstream target genes such as *AXIN2* and *LEF1* as well as a reduction in Dickkopf 1 (DKK1), which is the Wnt-induced Fzd-LRP 5/6 complex inhibitor (Semënov et al., 2001). Treatment with DKK1 then resulted in a reduction of Wnt transduction activity in the enriched CSCs while having no effect on the enriched normal breast stem cells. These findings clearly showed that canonical Wnt signaling pathway is differentially activated only in CSC but not normal stem cells. Increased canonical Wnt signaling activity was also detected in the side population (SP) of colon cancer cells sorted by Hoechst dye efflux, namely one of the assays applied to isolate CSC, as compared to non-SP cells. The latter study showed that β-catenin/TCF responsive luciferase reporter activity and expression of *C-MYC*, which is an important target gene of the pathway, were highly increased in SP cells than in non-SP cells (Chikazawa et al., 2010).

All these recent findings implied the critical roles of canonical Wnt signaling pathway in cell regulation in normal condition of the human body as well as in cancer development through regulation of cell expansion and survival of both mature and stem cells by their direct and/or indirect target genes (**Table 2**). This pathway has been demonstrated in many studies as a regulator of CSC traits in several cancers such as breast, colon, lung, pancreatic, and head and neck cancers, thus suggesting its critical roles in tumor recurrence. In this review, the roles of the pathway in regulating the stemness properties of CSC in a common type of head and neck cancer, which is HNSCC, will be further discussed in details.

**Table 2:** Elevated expression of upstream and downstream of Wnt signaling pathway genes and their associated diseases.

Gene transduction level	Gene	Disease (cell type)	Associated biological function of the gene	Reference
Upstream	*WNT*	Head and neck squamous cell carcinoma (cancer stem cell)	Self-renewal	Lee et al., 2014
	*FZD*			
Downstream	*AXIN2*	Breast cancer (cancer stem cell)		Lamb et al., 2013
	*LEF1*			
	*C-MYC*	Colon cancer (cancer stem cell)		Chikazawa et al., 2010
	*NANOG*	Non-disease (embryonic stem cell)		Zhang and Wang, 2008
	*SOX2*			
	*OCT4*	Non-disease (embryonic stem cell)		
		Head and neck squamous cell carcinoma (cancer stem cell)		Lee et al., 2014
	*MMP7*	Oral squamous cell carcinoma (cancer cell)	Metastasis	Iwai et al., 2010
	*TWIST*	Breast cancer (immortalized human breast epithelial cell)		Onder et al., 2008
		Head and neck squamous cell carcinoma (cancer cell)		Way et al., 2014
	*ABCB1*	Colon cancer, neuroblastoma (cancer cell)	Drug resistance	Yamada et al., 2000; Flahaut et al., 2009; Chikazawa et al., 2010


## The Roles of Canonical Wnt Signaling Pathway in the Regulation of HNSCC CSC Properties

### Self-Renewal and Differentiation

Replenishment of dead cells through differentiation of stem cells into specific mature cells is important for normal body function. This process is closely related to self-renewal of stem cells whereby continuous formation of mature cells is only possible when the long-lived stem cells constantly maintain their total cell number through cell proliferation. Tissue injury and diseases, such as cancer, promote remarkable changes in total stem cell number, thus suggesting that the balance between self-renewal and differentiation relies on the physiological needs of the body (Macdonald et al., 2004). Yet again, it is important to note that the molecular mechanisms that regulate the balance between both activities are still poorly understood but accumulating evidence suggested that stem cell behavior is regulated by a combination of cell-intrinsic signals as well as extrinsic signals from the stem cell niche (Li, 2006). In early embryonic development, multiple activation of extrinsic signaling molecules from the niche including fibroblast growth factor (FGF), transforming growth factor β (TGFβ), activin and nodal promote the increase in transcription factors, including Nanog, Oct4, and Sox2, which are important for the self-renewal of embryonic stem (ES) cells. In contrast, inhibition of these transcription factors through activation of BMP4 directs ES cells to differentiate (Zhang and Wang, 2008). Many other extrinsic signaling molecules are involved in the control of stem cell number and differentiation, and they include Hedgehog, Notch as well as Wnt, thus marking the importance of canonical Wnt signaling pathway in stem cell regulation (Li, 2006). Other than that, it has been recently proposed that regulation of the balance between self-renewal and differentiation of stem cells depends on c-Myc, a key mediator for the interaction of HSC and their niche whereby it modulates the migration and/or adhesion of HSC to the niche (Macdonald et al., 2004).

Canonical Wnt signaling pathway has been reported to play a critical role in the HSC proliferation, thereby promoting normal HSC homeostasis *in vitro* as well as *in vivo* (Reya et al., 2003). The importance of the activity of canonical Wnt signaling pathway has also been described in other types of stem cells such as epidermal and intestinal epithelial stem cells. It has been suggested that dysregulation of canonical Wnt signaling pathway which controls the self-renewing mechanism of stem cells promotes hyperproliferation of phenotypically similar daughter cells as observed in leukemia as well as intestinal and skin cancers (Pardal et al., 2003; Li, 2006).

Recently, the role of canonical Wnt signaling pathway in regulating self-renewal of HNSCC CSC is also being emphasized in several studies. Abnormal activation of the pathway has been correlated with increased proliferation and thus self-renewal of CSC in HNSCC. In oral squamous cell carcinoma (OSCC), CSC proliferation rate has shown to be influenced by the modulation of canonical Wnt signaling pathway through Wnt activator, 6-bromoin-dirubin-3′-oxime (BIO) and Wnt inhibitor, DKK1. Neither BIO nor DKK1 showed significant differences in its proliferative effects in parental OSCC cells, suggesting that the influence of the pathway on cell proliferation is limited to CSC only (Felthaus et al., 2011). Increased proliferation of HNSCC CSC caused by abnormal activation of the pathway can be indicated by β-catenin overexpression along with elevated expression of upstream components such as Wnt- and Fzd-encoding genes (Lee et al., 2014). Analysis on CSC proliferation upon stimulation by inhibitors of canonical Wnt signaling pathway has become a recent experimental approach in order to correlate the role of the pathway in self-renewal of CSC. In nasopharyngeal carcinoma (NPC), treatment of CSC isolated from HNE1 cell lines with Wnt-C59, a Wnt inhibitor, has been shown to reduce the proliferation of the CSC (Cheng et al., 2015). Furthermore, several other studies have also demonstrated a reduction in expression of β-catenin and eventually a suppression in the proliferation of CSC in HNSCC by several inhibitors of canonical Wnt signaling pathway including secreted frizzled-related protein 4 (sFRP4), all-trans-retinoic acid (ATRA) and honokiol, an active natural compound (Lim et al., 2012; Yao et al., 2013; Warrier et al., 2014). These findings clearly illustrated that canonical Wnt signaling pathway is responsible for the self-renewal capabilities of CSC in HNSCC. Hence, the understanding of molecular mechanisms by which this specific pathway regulates self-renewal of HNSCC CSC is crucial for improving treatment and prognosis. Recent study has suggested that the pathway regulates self-renewal of HNSCC CSC via the involvement of its downstream Oct4 activation. Silencing of β-catenin resulted in decreased Oct4 expression as well as self-renewing capacity of CSC, and subsequent ectopic expression of Oct4 in those shβ-catenin HNSCC CSC restored tumor sphere formation (Lee et al., 2014).

Furthermore, canonical Wnt signaling pathway also plays a major role in regulating stem cell differentiation during the development of early embryonic (Li, 2006; Vlad et al., 2008) as well as in cancer including HNSCC. It has been reported that this pathway was highly activated in CSC isolated from HNSCC M3a2 and M4e cell lines, and when injected into nude mice, these CSC differentiated into tumor cells and eventually resulted in five times larger tumor growth after 8 weeks compared to non-CSC. Thus, these signified the correlation of canonical Wnt pathway with differentiation and tumorigenicity of CSC (Song et al., 2010). Recent study has also proposed that cell differentiation of NPC CSC is regulated through the activity of canonical Wnt signaling pathway in the tumor microenvironment. The latter proposition was based on the finding that Wnt inhibitor, Wnt-C59, suppresses canonical Wnt signaling pathway in the tumor microenvironment rather than in the tumor cells themselves, thus resulted in suppression of the formation of growing tumors *in vivo* when induced with HNE1 cells which presumably contains CSC. In contrast, the control group without the inhibitor formed progressive growing tumor rapidly after a long latency period (Cheng et al., 2015).

In addition, the regulation of CSC differentiation by canonical Wnt signaling pathway has been recently exploited to overcome treatment failure. For instance, it is suggested that treatment failure and relapse in cancer could mainly be attributed to the major characteristic difference in chemosensitivity of CSC and differentiated cancer cells. Therefore, it is hypothesized that induced differentiation therapy may provide a solution for stem cell disease including cancer (Li, 2006; Zhang and Wang, 2008). Use of ATRA to suppress β-catenin expression, induced the conversion of CSC into differentiated HNSCC cells with reduced drug resistance capacity (Lim et al., 2012).

All these evidences clearly emphasized the roles of canonical Wnt signaling pathway in mediating both self-renewal and differentiation of CSC in HNSCC. However, to date, the regulation mechanisms of Wnt signaling pathway on both these properties are still not clearly understood; thus further studies are needed.

### Cell Metastasis

The underlying mechanisms of cancer invasion and metastasis have been well-described to be mediated by epithelial-mesenchymal transition (EMT) process. In cancer, EMT process involves the disruption of cell adhesion and loss of cell polarity which eventually allows cells to migrate from the primary tumor, metastasize to distant sites, and undergo mesenchymal-epithelial transition (MET) at the metastatic sites in the body (Way et al., 2014). Several signaling pathways have been associated with the regulation of EMT in cancer progression and metastasis including Akt, Hedgehog, Notch as well as Wnt signaling pathways (Lamouille et al., 2014). In malignant tumor of epithelial origins, one of the features of EMT is the loss of E-cadherin, an important membrane bound glycoprotein for cell-cell adhesion, which promotes the destabilization of adherens junctions between cells and hence acquisition of cell invasiveness and metastatic properties (Takebe et al., 2010; Wang et al., 2010).

High expression of matrix metalloproteinases (MMPs), a family of enzymes that acts as proteolytic destructor of basement membrane and extracellular matrix (ECM) components including E-cadherin, is often correlated with the invasiveness and metastasis of cancer (Van Roy and Berx, 2008). The levels of MMP1, MMP2, and MMP9 were found to be highly expressed in immunohistochemistry analysis of tumor invasive front tissues of patients with HNSCC (Franchi et al., 2002). Furthermore, MMP9 was also highly expressed in CSC-containing basal-cell-like cell layer located at the invasive front of HNSCC tumor, but not in basal cell layer of normal mucosa, thus facilitating tumors invasion via degradation of ECM (Sterz et al., 2010). Intriguingly, elevated expression of MMPs is directly associated with the activation of canonical Wnt signaling pathway, hence suggesting the role of the pathway in regulating metastasis in tumor cells as well as CSC in HNSCC. This is supported by the finding of elevated *MMP7* gene expression in OSCC cells with highly activated canonical Wnt signaling pathway. The increase in MMP7 expression resulted in redistribution of E-cadherin and cytoskeleton changes, which are often observed in early EMT (Iwai et al., 2010).

Furthermore, canonical Wnt signaling pathway is also implicated in the metastasis process of cancer through regulation of genes that facilitate EMT. Particularly, the EMT-related genes encoding for transcription factors such as Snail1 (Lim et al., 2011; Way et al., 2014), Snail2 (Slug; Zheng et al., 2009) and Twist are highly expressed in activated canonical Wnt signaling pathway (Smith et al., 2013a). In general, the main role of these transcription factors is to repress E-cadherin expression by silencing the promoter of *CDH1*, which is the gene that encodes for E-cadherin (Onder et al., 2008). It has been reported that increased expression of Snail1 correlates with significant reduction in E-cadherin expression in CSC from HNSCC cell lines grown as spheroid, resulting in EMT induction and metastasis. The direct link of CSC metastasis and canonical Wnt signaling pathway has not been proven in the latter study, though it can be hypothesized that activated canonical Wnt signaling pathway could be one of the mechanisms implicated in the increase in EMT-related protein expression (Chen et al., 2011). This may be supported by *in vitro* study of immortalized human breast epithelial (HMLE) cells whereby the activation of canonical Wnt signaling pathway promotes the increase in EMT-related protein expression, particularly Twist, and eventually contributes to cell metastasis through the loss of E-cadherin (Onder et al., 2008). In HNSCC, emodin, a compound from rhubarb plant, showed a significant inhibitory activity on Twist, evidenced by the decrease in β-catenin accumulation. This in turn results in the suppression of canonical Wnt signaling pathway and eventually the repression of EMT in HNSCC FaDu-pFLAG-*TWIST1* cells in *in vitro* as well as *in vivo* studies (Way et al., 2014). All these findings clearly emphasized the involvement of canonical Wnt signaling pathway in contributing cell invasiveness and metastasis in tumor cells as well as CSC of HNSCC.

In spite of this, the regulation of cell migration and invasion through EMT via non-canonical Wnt signaling has also been observed in several other metastatic cancers. In melonama,Wnt5A/Protein Kinase C pathway, independent of β-catenin, is highly activated and overexpression of EMT markers such as Snail and vimentin has been observed (Dissanayake et al., 2007). Furthermore, a recent study has discovered that a new, non-canonical Wnt signaling pathway mediated by Fzd2 receptor and Wnt5 ligands (Wnt5A and Wnt5B) induces overexpression of EMT markers such as MMP2 and MMP9 in several malignancies such as lung, colon, liver, and prostate cancers. Activation of Wnt5-Fzd2 signaling promotes Stat3 phosphorylation and activation mediated by tyrosine kinase Fyn (Ma et al., 2014). Co-regulation and independent regulation of both canonical and non-canonical Wnt signaling pathway in cancer metastasis have also been shown as recombinant Wnt5A further increased the mammosphere formation in *ex vivo* grown epithelial cells from *Wnt1*-overexpression mice. Instead of further activation of Wnt/β-catenin signaling pathway, the recombinant Wnt5A mediates non-canonical Wnt signaling pathway via receptor tyrosine kinase Ror2 and JNK signaling (Many and Brown, 2014). However, neither the independent nor co-regulation role of both canonical and non-canonical Wnt signaling pathways has been conclusively demonstrated in HNSCC.

### Drug Resistance

The major factor of recurrence in HNSCC is due to the failure of conventional chemotherapy to eliminate the critical CSC, allowing them to initiate recurring tumor cells which are more resistant and aggressive (Yanamoto et al., 2014). Many studies reported that the canonical Wnt signaling pathway is one of the underlying mechanisms in mediating the chemoresistance capacity of CSC in HNSCC. A naturally occurring Fzd/Wnt antagonist, sFRP4, has demonstrated an increase in the sensitivity of HNSCC CSC to cisplatin through inhibition of canonical Wnt signaling pathway, whereby combinative treatment of sFRP4 and cisplatin showed a significant reduction in CSC viability to 25% lesser than treatment with cisplatin alone. Moreover, the study also found that sFRP4 acts specifically on HNSCC CSC with dysregulated canonical Wnt signaling pathway whilst having no effect on non-cancerous cells with low level of the pathway activity. Therefore, this study greatly implied the high correlation of canonical Wnt signaling pathway and drug resistance in HNSCC CSC (Warrier et al., 2014).

The molecular mechanism by which canonical Wnt signaling pathway promotes chemoresistance properties in CSC is currently not well-defined. However, accumulating evidence suggested that ABC family transporter proteins mediate the resistance of CSC, derived from several cancer cell lines and primary tumors, toward a great number of conventional therapeutic drugs as well as radiotherapy (Cojoc et al., 2014). These proteins assist in therapeutic drugs efflux from cells thus contributing to anti-apoptotic effects (Chen et al., 2013). The most studied ABC family transporter protein subtype involved in drug efflux of HNSCC CSC is ABCG2, whereby its high expression correlates with marked chemoresistance and is mainly associated to the increase in canonical Wnt signaling pathway activity in HNSCC CSC (Zhang et al., 2009; Song et al., 2010; Yanamoto et al., 2011; Yao et al., 2013; Warrier et al., 2014).

Besides, several other subtype of ABC family transporter proteins, such as ABCC1, ABCC2, ABCC3, ABCC4, and ABCC5, were shown to be highly expressed in HNSCC spheroid cells with hyperactivated canonical Wnt signaling pathway. In addition, the latter study found that the knockdown of β-catenin in the spheroid cells suppressed their self-renewal capacity, chemoresistance, as well as expression of stem cell markers including Oct4, Sox2, CD44, and the ABC family transporters (Lee et al., 2014). Although the link of canonical Wnt signaling pathway and ABC family transporter protein for drug resistance capacity has been demonstrated in many studies, the molecular basis whether the pathway directly regulates the transcription of ABC family transporter proteins in HNSCC remains to be elucidated. Several studies which investigate the association of activated canonical Wnt signaling pathway and overexpression of ABC family transporter proteins in other types of cancer, such as colon cancer and neuroblastoma, have suggested that MDR1, also known as ABCB1, a subfamily B of ABC family transporter proteins, is a protein product from one of the direct target genes in the pathway (Yamada et al., 2000; Flahaut et al., 2009; Chikazawa et al., 2010). These accumulating discoveries may provide insight that canonical Wnt signaling pathway could also be one of the mechanisms that directly regulate the transcription of ABC family transporter proteins for chemoresistance capacity in HNSCC CSC.

## Druggable Targets in Wnt Signaling Pathway

Until recently, a wide range of compounds which inhibit dysregulated Wnt signaling activity in cancer have been discovered through successful preclinical studies and they include biosynthetic small molecule drugs, existing drugs as well as natural compounds (Lee et al., 2011; Le et al., 2015). Designing biosynthetic small molecule inhibitors which specifically target proteins interactions or enzymatic activities in Wnt signaling pathway has recently become an alternative strategy for discovery of selective Wnt pathway inhibitors. In this approach, it is crucial to understand the molecular mechanisms of the pathway to explore potential druggable targets (Verkaar and Zaman, 2011).

One of the recent approaches is by suppressing excessive Wnt signaling pathway activation using inhibitors against enzymatic activity of Porcupine (PORCN), which is an enzyme necessary for Wnt palmitoylation, secretion and biologic activity. For instance, inhibition of PORCN leads to reduction in palmitoylation of serine residue of Wnt to suppress Wnt secretion and its binding interaction to Fzd (Proffitt et al., 2013). In recent study, the effect of PORCN inhibitor, LGK974, had been examined in rodent models of Wnt-dependent breast tumor and human HNSCC HN30 cell line (Liu et al., 2013). Furthermore, other PORCN inhibitors such as Wnt-C59 and ETC 159 have also been studied for their effects against mammary tumors in MMTV-Wnt1 transgenic mice (Proffitt et al., 2013; Madan et al., 2015).

*In vivo* studies of those PORCN inhibitors have shown significant tumor growth regression with evident downregulation of Wnt target genes. Furthermore, the toxicity study also displayed no pathological changes in other normal Wnt-dependent tissues, including the intestine, stomach, brain, and skin, with no significant body weight loss at effective and well-tolerated dose of those inhibitors. In contrast, a very high dose promotes excessive inhibition of Wnt signaling pathway and causes significant effects to those Wnt-dependent tissues, including alopecia, intestinal damage, body weight loss, neurologic impairment, and increased neutrophils secondary to inflammation (Liu et al., 2013; Proffitt et al., 2013; Madan et al., 2015). Furthermore, the treatment of Wnt-C59 against bone marrow niche in MMTV-Wnt1-driven mouse mammary tumor models exhibited no toxicity on HSC with no significant effect on complete blood cell count, thus suggesting Wnt secretion can be suppressed using PORCN inhibitor without affecting normal adult hematopoiesis (Kabiri et al., 2015). It may be speculated that less sensitivity of normal tissues to modulation of Wnt-C59 compared to Wnt-driven tumor might due to their adaptation to low Wnt signals and/or they have alternative pathways for self-renewal (Proffitt et al., 2013). In addition, another recent finding demonstrated that LGK974 showed delayed pharmacodynamics with a significant time delay between peak drug concentration and maximum Axin2 mRNA inhibition. The delayed pharmacodynamics might be due to the mechanism of action of the drug which only blocks the secretion of newly formed Wnt while having no effect on pre-existing Wnt. Therefore, it is hypothesized that PORCN inhibitors are safe and effective in targeting tumor cells with excessive Wnt secretion compared to normal cells (Liu et al., 2013; Proffitt et al., 2013).

Furthermore, another biosynthetic enzyme inhibitor, XAV939 against tankyrase (TNKS), a regulator enzyme for Axin homeostasis, had been studied in colorectal cancer. XAV939 causes upregulation of Axin level and prolongs the half-life of Axin. An increase in Axin, which is the concentration-limiting component of the destruction complex in canonical Wnt signaling pathway, stimulates β-catenin degradation and eventually diminishes the pathway activation (Huang et al., 2009). Besides the enzymatic activities involved in Wnt signaling pathway, protein interactions in the pathway could also be one of the druggable targets. The interaction of β-catenin and T-cell factor 4 (TCF4), a transcription factor involved in canonical Wnt signaling pathway can be disrupted by PNU74654 drug as it forms a tight binding to β-catenin residue around K435 and R469, which is also the site of interaction with TCF4. The disruption of these proteins interaction eventually leads to inactivation of canonical Wnt signaling pathway (Trosset et al., 2006).

Other than that, several non-steroidal anti-inflammatory drugs, including aspirin and indomethacin, commonly used for treatment of pain, inflammation and fever, have been reported to inhibit the oncogenic canonical Wnt signaling pathway activity. It has been reported that indomethacin reduces canonical Wnt signaling pathway activity in a time- and dose-dependent manner through phosphorylation of NH2-terminal serine/threonine residues of β-catenin in colorectal cancer cells (Dihlmann et al., 2003). Aspirin also in a dose-dependent manner increases the phosphorylation of Tyr307 on the catalytic subunit of PP2A, namely a protein that regulates β-catenin dephosphorylation. This subsequently causes inactivation of the enzymatic activity of PP2A that will in turn promote increased β-catenin phosphorylation and eventually suppression of canonical Wnt signaling pathway activity (Bos et al., 2006).

Searching efforts for Wnt signaling pathway inhibitors among natural products have led to several successful discoveries. In osteosarcoma cells, curcumin, the active ingredient of turmeric, demonstrated a significant suppression of extrinsic activation of β-catenin/TCF transcriptional activities using either GSK-3β inhibitor (BIO), wild type β-catenin plasmid or mutant S33Y β-catenin plasmid (containing gene of mutant β-catenin which is resistant to Axin-APC-GSK3β destructive complex) in a dose dependent manner. Furthermore, they also found that the amount of nuclear β-catenin was reduced while no changes in the level of cytoplasmic β-catenin were seen after curcumin treatment. These findings suggested that the action of curcumin is independent of Axin-APC-GSK3β multiprotein destructive complex machinery. Rather, it involves the inhibition of nuclear translocation of β-catenin for further β-catenin/TCF transcriptional activities. However, the interaction of curcumin and β-catenin was not further investigated in the study (Leow et al., 2010, 2014). Other than that, it is reported that epigallocatechin-3-gallate (EGCG), the major polyphenol in green tea, also demonstrated potential anticancer effects against lung cancer cells through reactivation of the methylation-silenced gene Wnt inhibitory factor 1 (*WIF1*). Subsequently, this will restore the expression of WIF1 protein which acts as a Wnt antagonist by direct binding to Wnt molecules for downregulation of canonical Wnt signaling pathway (Gao et al., 2009).

Despite the increasing number of Wnt inhibitors, yet to date, no single inhibitor particularly targeting canonical Wnt signaling pathway has been approved to be used in clinical practice. Nevertheless, a Wnt pathway antagonist namely ipafricept OMP54F28, a recombinant fusion protein consisting of the ligand-binding domain of human Frizzled 8 (Fzd8) receptor and human IgG1 Fc fragment, is advancing into the first phase of clinical trial. The binding of OMP54F28 to Fzd8 prevents binding of Wnt to its receptor for downstream Wnt signaling activation. Promising preclinical findings of OMP54F28 showed significant *in vitro* inhibition of canonical Wnt signaling pathway and eventually *in vivo* suppression of tumor growth in pancreatic cancer. OMP54F28 in combination with gemcitabine also showed synergistic reduction of CSC frequency in pancreatic cancer (Hoey, 2013). Ongoing clinical trials of OMP54F28 include Phase 1a for advanced solid tumor and three Phase 1b clinical trials using combination treatment of OMP54F28 with sorafenib in hepatocellular cancer, nab-paclitaxel and gemcitabine in pancreatic cancer, as well as paclitaxel and carboplatin in ovarian cancer. Only preliminary data from first-in-human Phase 1 study of OMP54F28 targeting the Wnt pathway in patients with advanced solid tumors has been reported. The findings demonstrated that the well-tolerated dose is 10 mg/kg and the most common Grades 1 and 2 adverse effects seen were decreased appetite, fatigue, hypocalcemia, nausea, vomiting, dysgeusia, increased blood pressure, and peripheral edema (Lee et al., 2011; Smith et al., 2013b; Yeung et al., 2014; Fischer et al., 2015).

Other than that, it has been reported that naturally occurring compounds, resveratrol and resveratrol-containing freeze-dried grape powder (GP), have been examined for their effects on Wnt signaling pathway in colon cancer and normal colonic mucosa of eight volunteer colon cancer patients in a phase 1 pilot clinical trial. No significant inhibitory effect on Wnt signaling pathway was seen in colon cancer tissue for both treatments. Nevertheless, GP with a low dose of resveratrol in combination with other bioactive compounds demonstrated inhibition of Wnt signaling in normal colonic mucosa as indicated by depletion in the expression of Wnt target genes such as *CYCLIN D1* and *AXIN2* as compared to a low dose of resveratrol alone. Hence, this finding suggested that resveratrol or resveratrol-containing GP could act as potential colon cancer preventive agents rather than treatment of colon cancer via targeting Wnt signaling pathway (Nguyen et al., 2009).

## Concluding Remarks and Future Perspectives

Promising findings of preclinical studies and early phase of clinical trial of targeted therapy against canonical Wnt signaling-driven tumor have led to new insight into attractive therapeutic interventions for targeting CSC which are also principally regulated by canonical Wnt signaling pathway. As discussed previously, the properties of CSC of HNSCC, such as cell self-renewal, differentiation, metastasis, and chemotherapy resistance, are mainly regulated by the abnormal canonical Wnt signaling pathway activity and hence implicated in the development and progression of HNSCC. Although the crosstalk between multiple signaling pathways plays the vital role in the development of cancer, further understanding of one important molecular mechanism, which is canonical Wnt signaling pathway, is critical as targeting the pathway could be a promising approach in eradicating the treatment failure and relapse in HNSCC.

As complex as cancer disease, it is important to note that targeting aberrant canonical Wnt signaling pathway is challenging. Several major obstacles of clinical drug development of Wnt inhibitors are due to either insufficient efficacy, unspecific binding target, or the concern of the therapeutic window of the inhibitors. For instance, the efficacy of inhibitors may be insufficient as a single-agent treatment or be limited by the low bioavailability. Thus, combination treatment with other drugs and development of a good drug delivery system can be a good approach to be applied. Furthermore, since Wnt signaling pathway is also essential for normal cell homeostasis, selective binding of inhibitors and eventually inhibition of the pathway limited to cancer cells is crucial in order to circumvent any unfavorable effects in human body. The other major concern includes the challenge in obtaining the range of doses that produces therapeutic response without causing significant toxicity in patients. Complex and profound study on the efficacy, safety, and toxicity of the candidate drugs is fundamental in pursuing a novel agent that potentially having low side effects and exhibits greater medical benefits outweigh the risks in the patient. Recently, drug repurposing efforts have becoming a prominent alternative to traditional drug discovery. Exploring other uses of the existing drugs is promising as tolerability and safety profiles of these drugs are already well established.

## Author Contributions

All authors listed, have made substantial, direct and intellectual contribution to the work, and approved it for publication.

## Conflict of Interest Statement

The authors declare that the research was conducted in the absence of any commercial or financial relationships that could be construed as a potential conflict of interest.
